# Percutaneous Aspiration (AngioVac) of a Mitral Valve Vegetation followed by a Transcatheter Mitral Valve-in-Valve Intervention

**DOI:** 10.14797/mdcvj.1104

**Published:** 2022-07-11

**Authors:** Jack Xu, Shravan Turaga, Jay Bhama, Srikanth Vallurupalli, Gaurav Dhar

**Affiliations:** 1 University of Arkansas for Medical Sciences, Little Rock, Arkansas, US

**Keywords:** endocarditis, valve-in-valve intervention, percutaneous aspiration

## Abstract

The AngioVac transcatheter aspiration system (Angiodynamics) is used to percutaneously extract thrombi as well as vegetations typically growing from the right heart. We report a case of a failed mitral stented bioprosthesis due to a large vegetation that was treated successfully with AngioVac evacuation through a transseptal puncture followed by valve-in-valve intervention in the same setting.

## Background

There are more than 20,000 mitral valve replacements performed annually in the United States.^1^ Most of these replacements are bioprosthetic valves, which have limited durability.^2^ Repeat operation on failed bioprosthetic mitral valves results in increased morbidity and mortality.^3^ Transcatheter mitral valve-in-valve (MViV) replacements using a transseptal (TS) approach have been increasing in popularity and are associated with high technical success and a low complication rate.^4^

In patients with high surgical risk, a minimally invasive technique that employs the AngioVac transcatheter aspiration system (Angiodynamics) is increasingly being used to debulk right-sided valve vegetations, thrombi, or tumors, with the goal of reducing the bacterial or embolic burden to temporize the acute illness.^5,6,7^ The AngioVac system is based on an extracorporeal circuit in a veno-venous configuration, and the system is rarely used in the left heart.^8^ We describe the novel use of simultaneous AngioVac evacuation and transcatheter MViV in the same procedure.

## Case presentation

**Figure 1:**
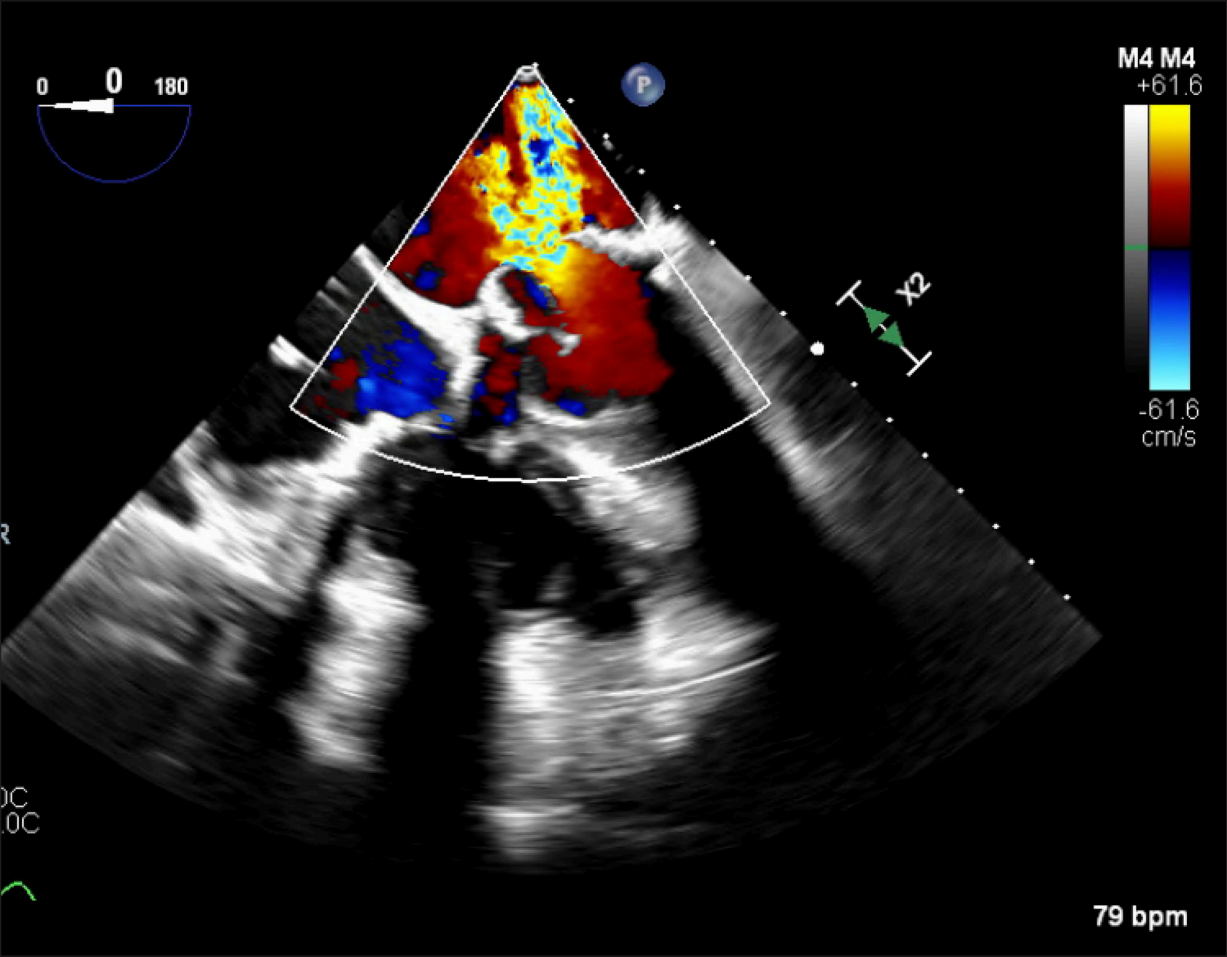
Transesophageal echocardiogram showing severe mitral regurgitation.

**Figure 2:**
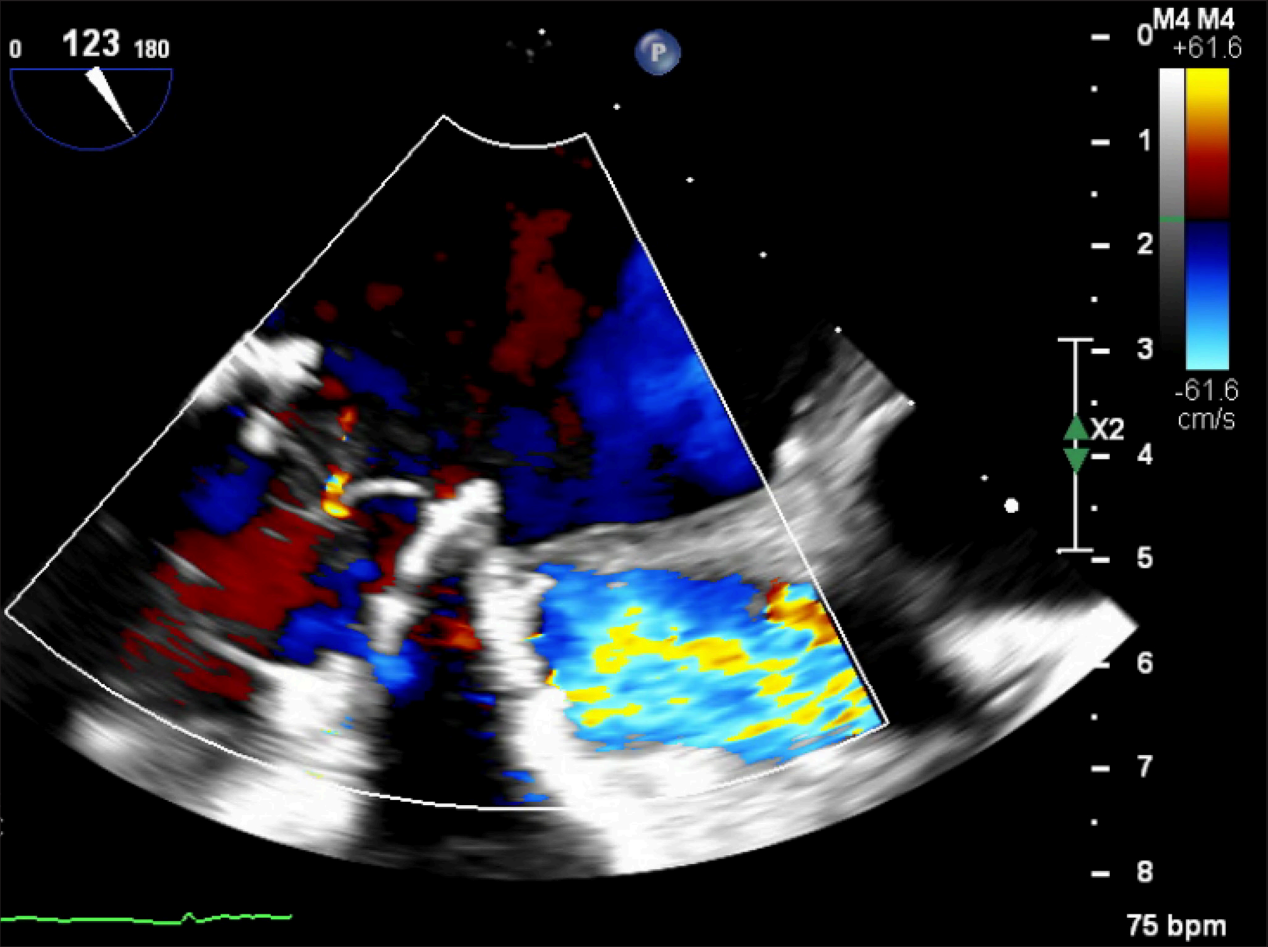
Transesophageal echocardiogram showing trivial mitral regurgitation after valve-in-valve procedure.

A 49-year-old female with a history of end-stage renal disease (ESRD) presented with new onset congestive heart failure 12 months after undergoing mitral (29 mm St Jude Epic) and aortic valve replacements for calcific valve disease. Transthoracic echocardiogram (TTE) showed focally thickened mitral prosthetic leaflets with increased transmitral flow (mean gradient of 12 mm Hg at a heart rate of 74 beats per minute with a pressure half time of 96 msec). Transesophageal echocardiogram (TEE) showed a large oscillating echodensity suggestive of a vegetation; it attached to the prosthetic mitral valve ring that crossed the valve plane (Figure 1, Videos 1 A, 1 B). She had severe mitral regurgitation with a vena contracta of 0.9 cm (Figure 2). The aortic bioprosthesis displayed normal function. Blood cultures were obtained, and intravenous antibiotics were started. Based on her clinical presentation, history of flu-like illness 3 months prior to presentation, and negative blood cultures, this was determined to be an old vegetation.

**Video 1(A):** Transesophageal echocardiogram 2-dimensional video showing a large oscillating echodensity on the bioprosthetic mitral valve, also at https://youtu.be/hm8LprvaWDY.

**Video 1(B):** Transesophageal echocardiogram 3-dimensional video of oscillating echodensity, also at https://youtu.be/qfxDc736LG4.

The patient was deemed to be at prohibitive surgical risk for a redo mitral valve surgery because her Society of Thoracic Surgeons risk score was found to be 10.5% for mitral valve repair and 15.6% for mitral valve replacement. After a multidisciplinary team discussion and shared decision making with the patient, the decision was made to undergo extracorporeal aspiration of the mitral valve vegetation using the AngioVac system as well as a percutaneous transcatheter MViV replacement. The patient was treated with antibiotics and anticoagulation for several weeks prior to the procedure in an attempt to stabilize the vegetation as much as possible. Blood cultures were negative prior to the MViV.

**Figure 3:**
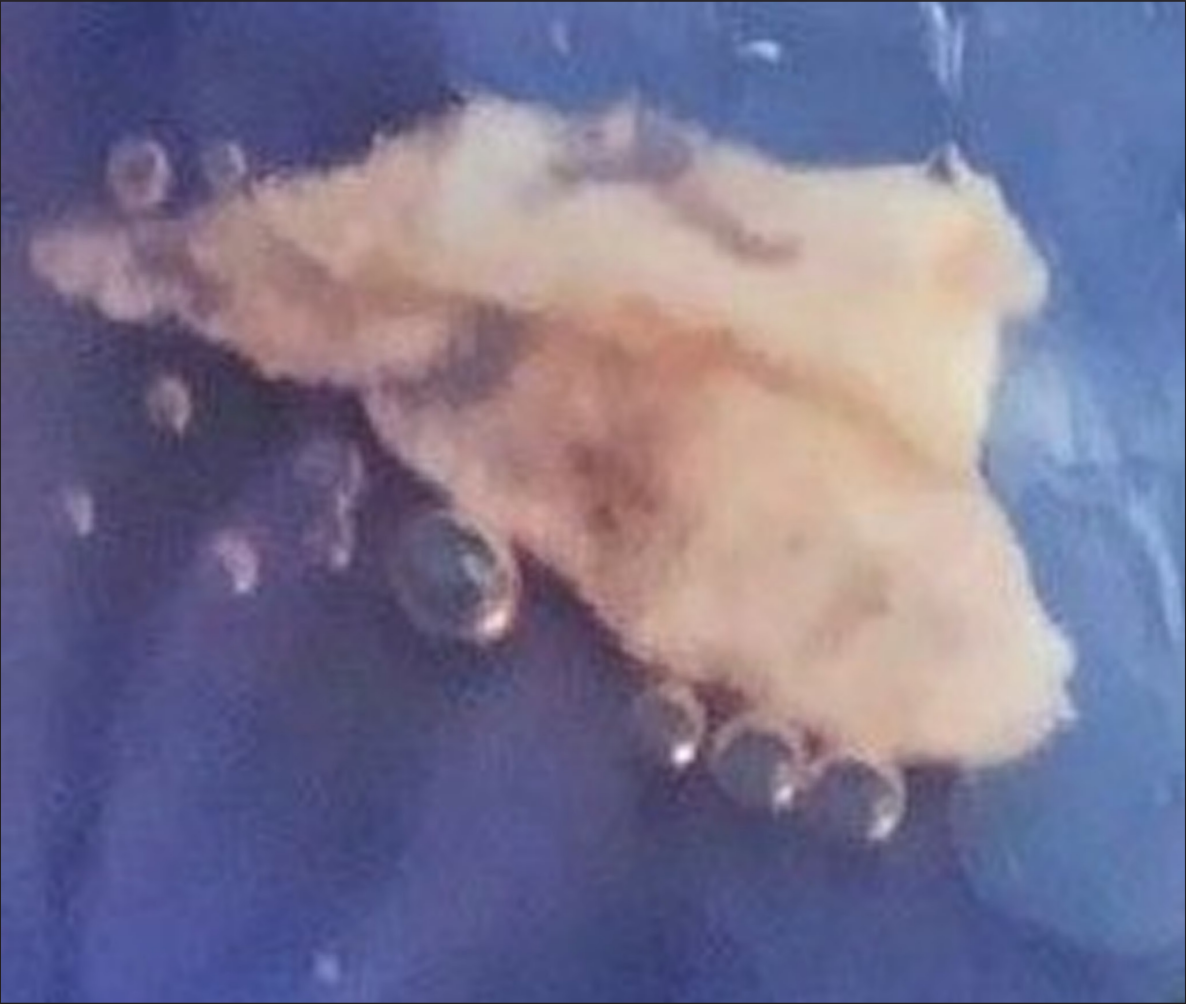
Aspirated material after AngioVac removal.

Due to the risk of thromboembolism and stroke during AngioVac of left-sided endocarditis, we considered using a SENTINEL™ Cerebral Protection System (Boston Scientific Corp.) but were unable due to access issues in the setting of her atrioventricular fistula. Under general anesthesia, a transseptal puncture was performed using a right femoral vein approach. The left femoral vein was also cannulated for the return of blood. Afterwards, the AngioVac cannula was advanced into the left atrium with the cannula directed towards the vegetation at the posterior aspect of the valve. The residual vegetation was carefully evacuated (Figure 3, Video 2). Then, an Agilis HisPro™ steerable catheter (Abbott) was used, and the bioprosthetic mitral valve was crossed using a JR 4 and J wire. A 26-mm Edwards S3 prosthesis was implanted through the same transeptal puncture site (Video 3). There was no residual vegetation, and the mean transmitral gradient after MViV implantation was 5 mm Hg (Video 4). The vegetation (Figure 3) was sent for culture and subsequently showed no growth. The iatrogenic atrial septal defect was not closed, which is typical unless special circumstances arise. After the procedure, the patient was treated with vancomycin, ceftazidime, and doxycycline for a 6-week course. She was not discharged on anticoagulation due to her bleeding risk and especially given her ESRD. She was eventually discharged home with resolution of her symptoms. One month after the procedure, TTE showed no residual mitral regurgitation (Video 5).

**Video 2:** Transesophageal echocardiogram video showing AngioVac removal of the vegetation, also at https://youtu.be/HJ1yQsG7wPU.

**Video 3:** Transesophageal echocardiogram video showing successful deployment of a 21-mm Edwards Trifecta™ Bioprosthetic Valve in the bioprosthetic mitral valve, also at https://youtu.be/qvsrElMhhKs.

**Video 4:** Transesophageal echocardiogram 3-dimensional video showing final results, also at https://youtube.com/shorts/vG34eVLoP5M.

**Video 5:** Transthoracic echocardiogram 1 month after valve-in-valve procedure, also at https://youtu.be/CqxZ1Pfmt-o.

## Discussion

This case was challenging since the patient was at prohibitive surgical risk, with a large vegetation that caused severe mitral regurgitation and a failing mitral bioprosthesis. Medical management alone was unlikely to resolve such a large vegetation or the severe mitral regurgitation. The initial AngioVac evacuation allowed safe implantation of the MViV prosthesis. Transcatheter aortic ViV implantation for aortic regurgitation secondary to bioprosthetic aortic valve endocarditis was reported by Fathi et al.^9^ They described a ViV transcatheter aortic valve implantation 8 months after treatment of infective endocarditis, and antibiotics were administered 6 weeks post procedure. The most common indications of ViV replacements include degenerated mitral prostheses, failed surgical rings, and severe mitral annular calcifications.^10^

A meta-analysis of studies using the AngioVac system has shown resolution of endocarditis in 80% of patients.^8^ Furthermore, a retrospective analysis of high-risk patients with a median Society of Thoracic Surgeons score of 10% who underwent MViV showed a 90.9% technical success rate and a 30-day mortality rate of 8.1%,^1^ with a left ventricular outflow tract obstruction rate of 0.7%. Post-procedure mitral valve function was good, with a median mitral valve gradient of 4 mm Hg and residual mitral regurgitation of 1+ or less in 98.1% of patients.^10^ However, the long-term effects of these procedures are not known and require further studies.

## Conclusion

This was a novel case of treatment for a failed mitral stented bioprosthesis using the AngioVac system to remove a large left-sided vegetation followed by a transcatheter MViV. Based on these findings, concomitant use of both procedures can be considered for large left-sided vegetations. While not approved by the US Food and Drug Administration for this indication, this combined approach may be an option for patients with high to prohibitive surgical risk.

## References

[1] Whisenant B, Kapadia SR, Eleid MF, et al. One-Year Outcomes of Mitral Valve-in-Valve Using the SAPIEN 3 Transcatheter Heart Valve. JAMA Cardiol. 2020 Nov 1;5(11):1245–1252. doi: 10.1001/jamacardio.2020.2974 32745164 PMC7391176

[2] Cheung A, Webb JG, Barbanti M, et al. 5-year experience with transcatheter transapical mitral valve-in-valve implantation for bioprosthetic valve dysfunction. J Am Coll Cardiol. 2013 Apr 30;61(17):1759–66. doi: 10.1016/j.jacc.2013.01.058 23500301

[3] Stone GW, Adams DH, Abraham WT, et al.; Mitral Valve Academic Research Consortium (MVARC). Clinical trial design principles and endpoint definitions for transcatheter mitral valve repair and replacement: part 2: endpoint definitions: a consensus document from the Mitral Valve Academic Research Consortium. J Am Coll Cardiol. 2015 Jul 21;66(3):308–321. doi: 10.1016/j.jacc.2015.05.049 26184623

[4] Guerrero M, Salinger M, Pursnani A, et al. Transseptal transcatheter mitral valve-in-valve: A step by step guide from preprocedural planning to postprocedural care. Catheter Cardiovasc Interv. 2018 Sep 1;92(3):E185–E196. doi: 10.1002/ccd.27128 28557344

[5] Joseph-Alexis J, Jaffe A, Jacinto JP, Akel R. AngioVac Removal of an Isolated Infected Pulmonary Valve Papillary Fibroelastoma. JACC Case Rep. 2020 Nov 18;2(14):2213–2216. doi: 10.1016/j.jaccas.2020.10.008 34317142 PMC8299978

[6] George B, Voelkel A, Kotter J, et al. A novel approach to percutaneous removal of large tricuspid valve vegetations using suction filtration and veno-venous bypass: a single center experience. Catheter Cardiovasc Interv. 2017 Nov 15;90(6):1009–1015. doi: 10.1002/ccd.27097 28471095

[7] Starck CT, Dreizler T, Falk V. The AngioVac system as a bail-out option in infective valve endocarditis. Ann Cardiothorac Surg. 2019 Nov;8(6):675–677. doi: 10.21037/acs.2019.11.04 31832358 PMC6892725

[8] Gerosa G, Longinotti L, Bagozzi L, et al. Transapical aspiration of a mitral mass with the AngioVac system on a beating heart. Ann Thorac Surg. 2020 Nov;110(5):e445–e447. doi: 10.1016/j.athoracsur.2020.04.051 32504600

[9] Fathi AS, Ali JM, Mann S, et al. Emergency valve-in-valve transcatheter aortic valve implantation for endocarditis degeneration. J Card Surg. 2020 Mar;35(3):713–715. doi: 10.1111/jocs.14441 31999375

[10] Guerrero M, Vemulapalli S, Xiang Q, et al. Thirty-day outcomes of transcatheter mitral valve replacement for degenerated mitral bioprostheses (valve-in-valve), failed surgical rings (valve-in-ring), and native valve with severe mitral annular calcification (valve-in-mitral annular calcification) in the United States: data from the Society of Thoracic Surgeons/American College of Cardiology/Transcatheter Valve Therapy Registry. Circ Cardiovasc Interv. 2020 Mar;13(3):e008425. doi: 10.1161/CIRCINTERVENTIONS.119.008425 32138529

